# Development of statistical regression and artificial neural network models for estimating nitrogen, phosphorus, COD, and suspended solid concentrations in eutrophic rivers using UV–Vis spectroscopy

**DOI:** 10.1007/s10661-023-11738-0

**Published:** 2023-08-31

**Authors:** Yanping Lyu, Wenpeng Zhao, Tsuyoshi Kinouchi, Tadahiro Nagano, Shigeo Tanaka

**Affiliations:** 1 Department of Transdisciplinary Science and Engineering, Tokyo Institute of Technology, 4259 Nagatsuta-Cho, Midori-Ku, Yokohama, Kanagawa 226-8503 Japan; 2 College of Hydraulic Science and Engineering, Yangzhou University, Yangzhou, 225009 China; 3 Civil Engineering and Eco-Technology Consultants Co., Ltd, 2-23-2 Higashi-Ikebukuro, Toshima-Ku, Tokyo, 170-0013 Japan

**Keywords:** Water quality monitoring, In situ UV–vis spectroscopy, Statistical regression models, Artificial neural network, Wavelength selection

## Abstract

River water quality monitoring is crucial for understanding water dynamics and formulating policies to conserve the water environment. In situ ultraviolet–visible (UV–Vis) spectrometry holds great potential for real-time monitoring of multiple water quality parameters. However, establishing a reliable methodology to link absorption spectra to specific water quality parameters remains challenging, particularly for eutrophic rivers under various flow and water quality conditions. To address this, a framework integrating desktop and in situ UV–Vis spectrometers was developed to establish reliable conversion models. The absorption spectra obtained from a desktop spectrometer were utilized to create models for estimating nitrate-nitrogen (NO_3_-N), total nitrogen (TN), chemical oxygen demand (COD), total phosphorus (TP), and suspended solids (SS). We validated these models using the absorption spectra obtained from an in situ spectrometer. Partial least squares regression (PLSR) employing selected wavelengths and principal component regression (PCR) employing all wavelengths demonstrated high accuracy in estimating NO_3_-N and COD, respectively. The artificial neural network (ANN) was proved suitable for predicting TN in stream water with low NH_4_-N concentration using all wavelengths. Due to the dominance of photo-responsive phosphorus species adsorbed onto suspended solids, PLSR and PCR methods utilizing all wavelengths effectively estimated TP and SS, respectively. The determination coefficients (*R*^2^) of all the calibrated models exceeded 0.6, and most of the normalized root mean square errors (NRMSEs) were within 0.4. Our approach shows excellent efficiency and potential in establishing reliable models monitoring nitrogen, phosphorus, COD, and SS simultaneously. This approach eliminates the need for time-consuming and uncertain in situ absorption spectrum measurements during model setup, which may be affected by fluctuating natural and anthropogenic environmental conditions.

**Supplementary Information:**

The online version contains supplementary material available at 10.1007/s10661-023-11738-0.

## Introduction

With the development of agriculture, industrialization, and urbanization, the deterioration of water quality in the natural environment is becoming a severe regional and global issue due to the increased loading of nutrients, organic carbon, and other toxic substances (de Waal et al., 2022; Ho et al., 2019; Rashid & Romshoo, 2013). Climate change will influence the water quality dynamics and ecosystem by increasing carbon dioxide concentration, air temperatures, more intense precipitations, etc. (Alexander et al., 2013). Water quality issues have been at the forefront of Sustainable Development Goals (SDGs), in which Goal 6 specifically aims to “ensure availability and sustainable management of water and sanitation for all” (UNESCO, 2015). Thus, water quality monitoring is increasingly important for sustainably managing water resources and maintaining ecosystem stability.

Water quality monitoring is a prerequisite for understanding the factors that drive the deterioration and for establishing water quality conservation and improvement goals. Traditional methods, such as manual water sampling and laboratory analysis of individual constituents in the samples, provide accurate data but are labor-intensive and inefficient, especially for frequent monitoring with short intervals. To address this, automatic instruments based on chemical reactions have been developed for real-time monitoring the water quality of streams, lakes, and wastewater (Bodini et al., 2018; Fang et al., 2019, 2022). However, these instruments generally require high maintenance costs and consume many chemicals. In the field of analytical chemistry and geochemistry, ultraviolet–visible (UV–Vis) sensors have emerged as efficient tools for analyzing soluble inorganic salts and organic compounds in water by recording the spectral absorbance (Birdwell & Engel, 2010; Willard et al., 1988). For operational purposes in the field rather than in a laboratory, the in situ UV–Vis spectroscopic technology has been developed rapidly and applied for continuous monitoring of the water environment under different hydraulic conditions (Pesántez et al., 2021; Zhang et al., 2022). For wastewater monitoring purposes, in situ UV–Vis spectrometer has been successfully applied to measure various parameters, including suspended solids (SS) and chemical oxygen demand (COD) (Brito et al., 2014; Langergraber et al., 2003). Several studies have explored the use of in situ UV–Vis spectrometers for monitoring nitrogen, carbon, phosphorus, and other components in different environments, such as springs and tidal marshes, by applying statistical models (Huebsch et al., 2015; Etheridge et al., 2014). However, there is limited research on their application in water bodies with diverse biogeochemical compositions, especially eutrophic rivers containing pollutants from farmlands, forests, and urban areas. Monitoring pollutant concentrations to obtain the loading from eutrophic rivers is increasingly important due to the nutrient enrichment and accelerating eutrophication in the receiving lakes (Ho et al., 2019; Izmailova & Rumyantsev, 2016; Smith et al., 1999).

The greatest challenge in applying in situ UV–Vis technology for monitoring stream water quality is establishing robust relationships between specific water quality parameters and UV–Vis absorption spectra under the conditions with complicated biogeochemical constituents in the stream. Interference from compounds with distinctive absorbance can lead to overestimated water quality parameters (Uusheimo et al., 2017). Moreover, high concentrations of suspended particles, particularly during flood events, significantly alter the absorbance signals due to light scattering and shading. Previous studies have attempted to estimate water quality parameters using absorption spectra by employing regression and neural network approaches (Etheridge et al., 2014; Peleato et al., 2018; Tong et al., 2019). However, their feasibility in eutrophic rivers, characterized by elevated levels of various compounds and suspended solids, remains uncertain. This uncertainty lies in identifying specific absorption bands that can establish relationships, especially for parameters such as COD and total phosphorus (TP), due to the presence of various compounds at different concentrations. Regression analysis methods such as principal component regression (PCR) and partial least squares regression (PLSR) are commonly used (Feudale et al., 2002), which can address the issue of multicollinearity by reducing the dimensionality of independent variables (wavelengths) (Wold et al., 1987). PCR and PLSR have advantages when the number of discrete samples is limited, but the number of independent variables (wavelengths) is substantial. Nevertheless, selecting a suitable subset of influential wavelengths from the spectra corresponding to each water quality parameter would improve model performance (Hemmateenejad et al., 2007). Compared with these linear regression approaches, artificial neural network (ANN), with its ability to describe nonlinear and complicated systems, may be more suitable for some specific water quality parameters (Tong et al., 2019). While ANN has been used for quantifying single parameters in wastewater monitoring (Fogelman et al., 2006; Li et al., 2020), its applications for estimating multiple water quality in streams remain limited.

Considering the challenges mentioned above and the limited previous studies for river water quality monitoring using in situ UV–Vis spectroscopy, this study aims to identify the most influential wavelengths for modeling various water quality parameters and find the effective and robust calibration methodology and models targeting eutrophic rivers characterized by high concentrations of organic and inorganic compounds as well as suspended sediments. Herein, we introduced a novel approach that integrates in situ UV–Vis technology with carefully designed modeling methods, including PCR, PLSR, and ANN, to accurately predict water quality parameters within a eutrophic watershed characterized by complex pollutant components and diverse land use conditions. The calibration process typically necessitates a substantial volume of data to include the broad range of concentrations exhibited by each water quality parameter, thereby requiring extensive fieldwork for data collection. Moreover, the inherent variability in flow and water quality parameters during the in situ spectrometer measurements can introduce uncertainties in the calibration data. To mitigate such uncertainties and reduce the labor-intensive nature of fieldwork associated with in situ spectrometers, we take full advantage of the absorption spectra obtained from sampled stream water in a controlled laboratory setting (consisting of 112 data samples) using a desktop UV–Vis spectrometer for calibration and validation purposes, employing several regression methods, i.e., PCR, PLSR, and ANN (as detailed in “Determination of the influential wavelengths” and “Selection of calibration methods” sections). Subsequently, these calibrated models are applied to absorption spectra measured in situ in rivers rather than in laboratory conditions using a portable in situ spectrometer (22 data samples), thereby further validating the applicability of this methodological framework and demonstrating the potential of in situ UV–Vis spectrometers for automated monitoring of water quality in natural environments, particularly eutrophic rivers (as discussed in “Water quality estimation results” section).

## Study areas and data source

### Study rivers

**Fig. 1:**
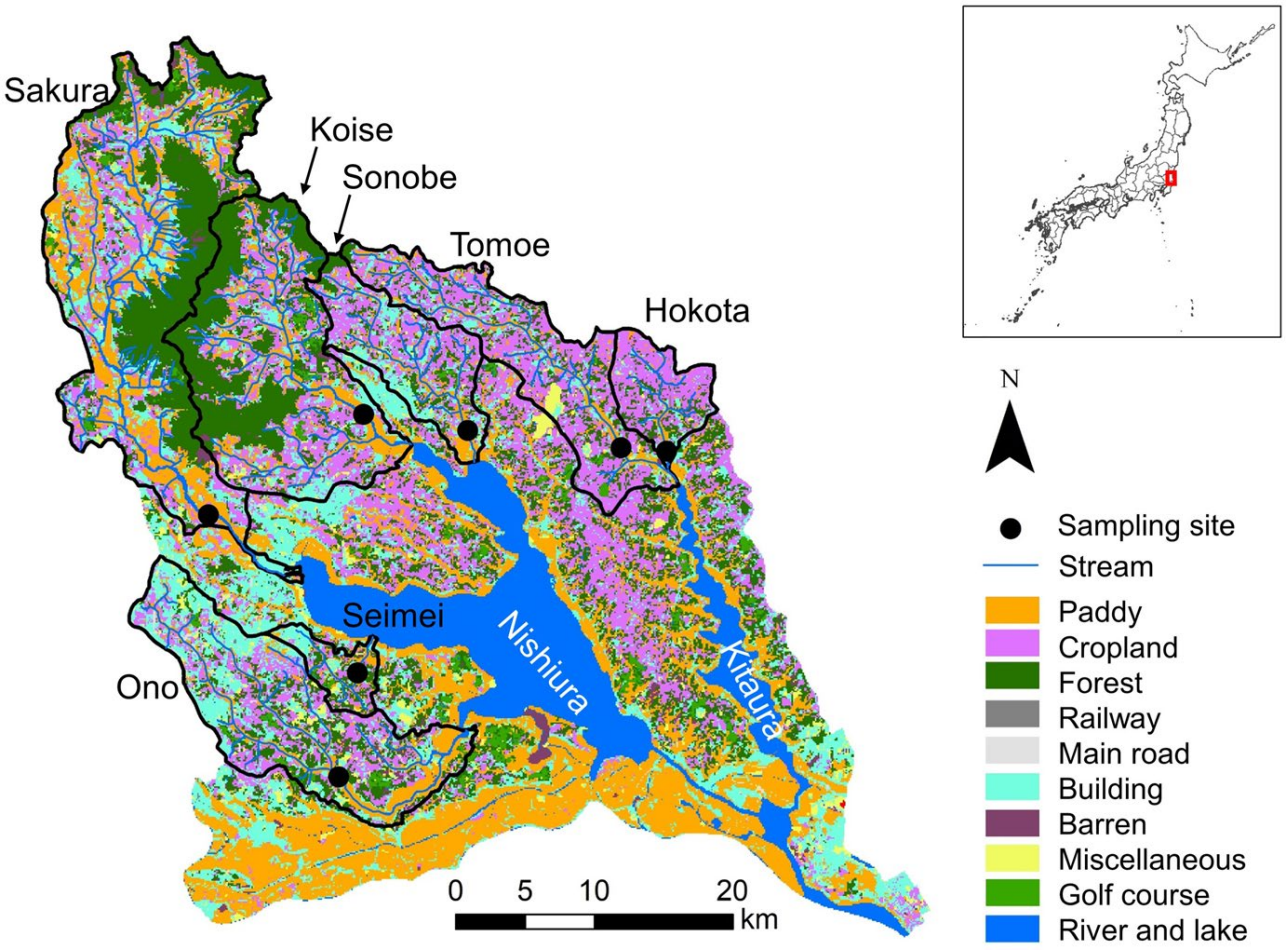
Location of sampling site in the mainstream of each watershed flowing into Lake Kasumigaura. The detailed land use types in the whole Kasumigaura Lake watershed are based on the GIS data developed by MLIT (Ministry of Land, Infrastructure, Transport and Tourism, Japan) using satellite images during 2016

The study rivers are located in the seven major watersheds of Lake Kasumigaura (Lake Nishiura and Lake Kitaura) with different land use characteristics (Fig. 1), all of which generate the surface and subsurface runoff and drain wastewater into the rivers and lakes. Lake Kasumigaura is the second largest lake in Japan, located in Ibaraki Prefecture, with a surface area of about 208 km^2^ and an average water depth of 4 m. Owing to the increase of nutrient loadings, significant water quality degradation, and frequent algae bloom in Lake Kasumigaura were reported until the 1980s. After that, no noticeable decrease in the nutrient concentrations was observed, while the concentration of TN has been increasing since the late 1990s. The entire watershed of Lake Kasumigaura, having a total area of 2157 km^2^ with a total population of about 0.98 million, is constituted by a mixture of different land use types such as paddy fields, croplands, forests, and buildings (Fig. 1). The proportion of paddy, cropland, forest, buildings, and other land covers in each of the seven watersheds are summarized in Table 1, which was obtained from the land use survey data for 2016 provided by the Ministry of Land, Infrastructure, Transport, and Tourism of Japan. Cropland and forest are the dominant land use types in the seven watersheds, occupying 28% of the area, respectively, and the paddy field occupies 20% of the area and is the third largest land use type. The sources of nutrients and carbons in these watersheds include agriculture (cropland and paddy), urban surface, mountain forests, animal husbandry, and treated and untreated domestic and industrial wastewater, which are all responsible for the current eutrophic water quality status in Lake Kasumigaura. The amount of loading from each watershed is different, depending on the specific condition of each source, in addition to the season and the hydrologic condition such as floods and low flows.

**Table 1:** Areal percentage of each land use type in each watershed

	Hokota	Tomoe	Sonobe	Koise	Sakura	Seimei	Ono	Seven watersheds
Total (km^2^)	44.7	129.5	81.9	222.4	335.0	24.9	176.3	1014.7
Paddy	11%	16%	18%	19%	27%	20%	17%	20%
Cropland	60%	50%	39%	25%	17%	23%	26%	28%
Forest	16%	17%	17%	40%	33%	25%	17%	28%
Buildings	10%	12%	22%	12%	15%	24%	27%	17%
Others	3%	6%	4%	5%	7%	9%	12%	7%

### Data source

The data used in this study can be divided into two parts: water quality data observed through chemical analysis and UV–Vis spectral data obtained through physical analysis. The flowchart of the data acquisition process is shown in Supplementary Fig. 1. For the analysis of water quality, water sampling was conducted in the tributary rivers of Lake Kasumigaura, i.e., the Hokota, Tomoe, Sonobe, Sakura, Koise, Ono, and Seimei Rivers (Fig. 1) during low-flow and flooding periods between 2016 and 2019. Water samples were analyzed in the laboratory to obtain the specific water quality parameters, including NO_3_-N, TN, COD, TP, SS, NH_4_-N, and DTP. All the water quality parameters were measured following the Japanese Industrial Standards (JIS) or Environmental Agency Ordinance (in the case of suspended sediment). COD was determined using the potassium permanganate (KMnO_4_) method. TN was quantified via the sodium hydroxide/potassium persulfate digestion method, coupled with copper cadmium reduction or UV spectrophotometry. NO_3_-N was determined using the copper-cadmium reduction method or ion chromatography. Ammonium ions were evaluated utilizing the indophenol blue colorimetric method. TP was measured using potassium persulfate digestion and absorbance spectrophotometry. For dissolved total phosphorus (DTP), the same method was used but preceded by filtration. SS was measured using filtration with a glass fiber filter with 1-μm pore sizes (ADVANTEC GA-100).

**Fig. 2:**
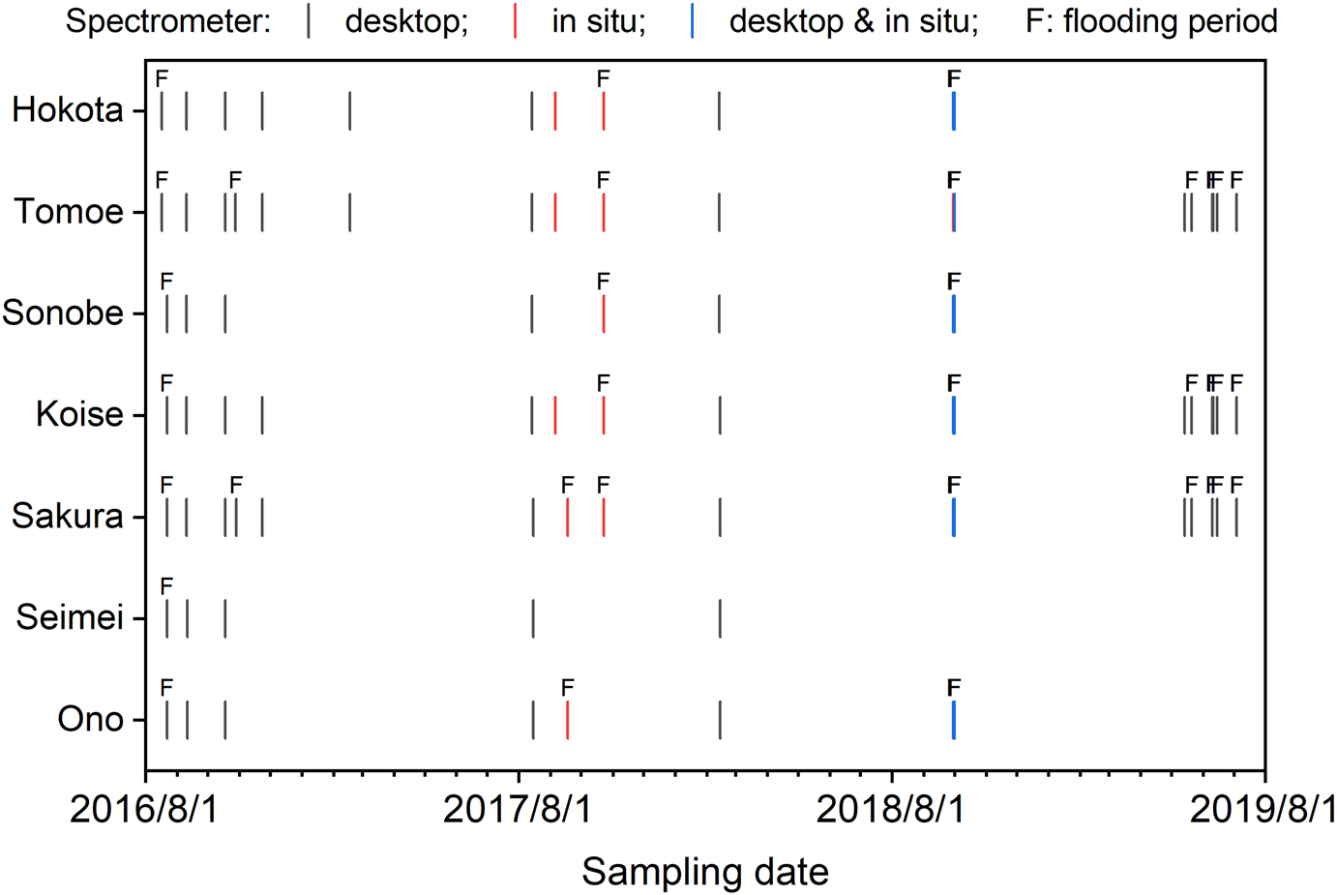
Dates and sites of water sampling and spectral measurement mode used

The absorption spectra corresponding to each water sample were measured in the field and/or in the laboratory (Fig. 2). In the field experiments, an in situ UV–Vis spectrometer (spectro::lyser, s::can Measuring Systems) with a 5-mm optical path length was placed in the center of the river, with a depth of ~ 0.2 m, at the sampling location to record the absorption spectra of the river water for 22 samples. The sweep range of the spectrometer is 220–735 nm, with 2.5-nm intervals. In the laboratory, a commonly used desktop spectrometer (Hitachi U2910) with a 10-mm optical path length was used to record the absorption spectra of 112 river water samples. The sweep range of the desktop spectrometer is 200–750 nm with 1-nm intervals. The laboratory data, characterized by a larger sample size, was used to determine the proper subset of influential wavelengths and establish robust models. In order to align the desktop and in situ UV–Vis data and ensure consistency, only the wavelengths divisible by 5 (i.e., 220–735 nm with a 5-nm interval) are valid for model calibration and validation.

## Methodology

### Research flow chart

**Fig. 3:**
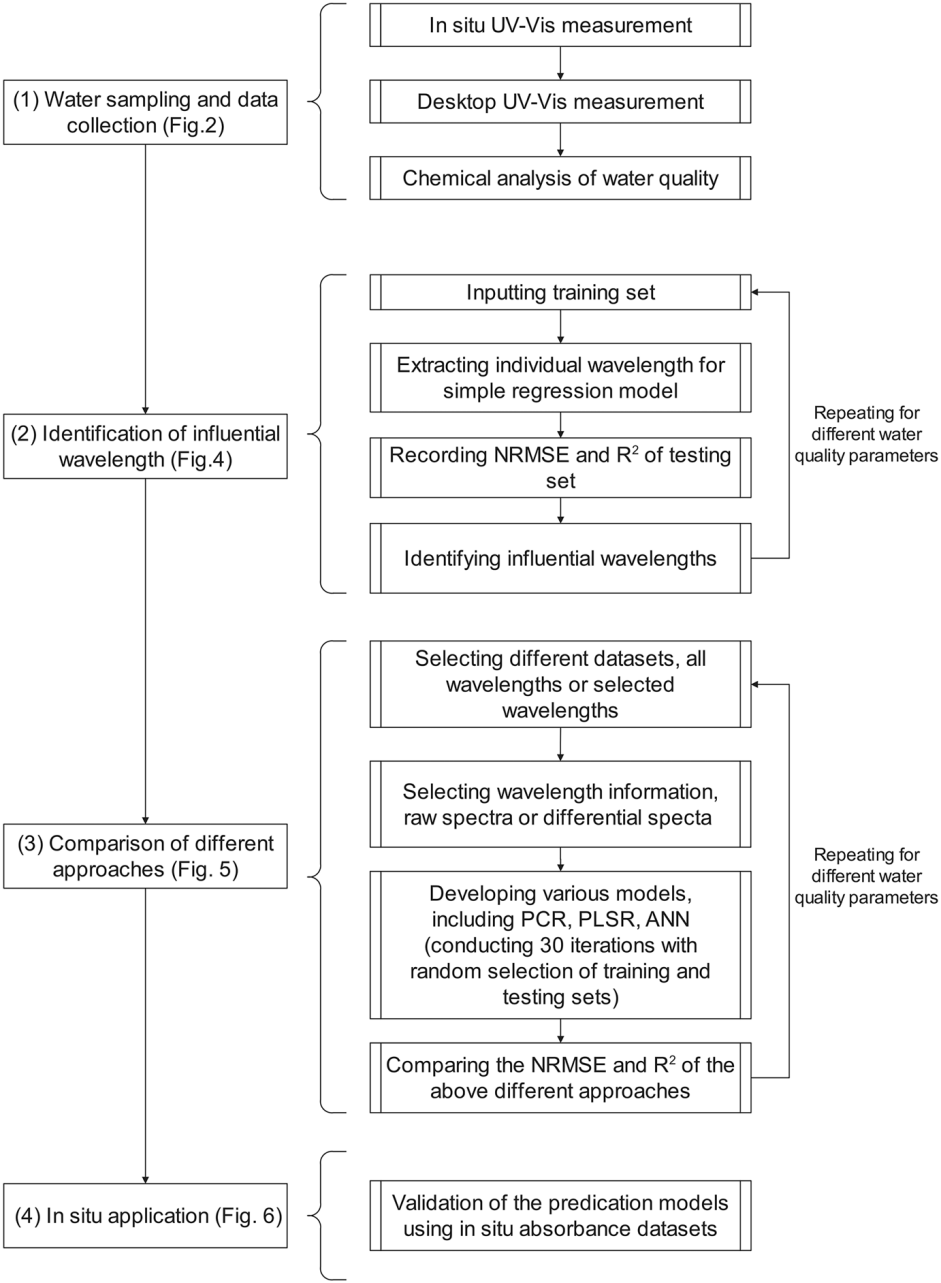
Summary of the research flow, modeling establishment, and validation processes

Figure 3 illustrates the flowchart outlining the methodology employed in this study and the detailed modeling establishment and validation processes. (1) We collected water samples, measured the UV–Vis spectra using the desktop mentioned above and/or in situ UV–Vis spectrometer (112 and 22 samples were measured by desktop and in situ spectrometer, respectively), and analyzed the water quality of each sample. (2) We selected the influential wavelengths by investigating the correlation between a specific water quality parameter and the UV–Vis absorbance at each wavelength from 220 to 735 nm. (3) We comprehensively compared the modeling approaches involving different regression or machine learning models, various subsets of wavelengths, and different pretreatment methods applied to the spectra. (4) We summarized the successful approaches and validated the performance of water quality estimation using the spectra acquired in the rivers using the in situ UV–Vis spectrometer. To realize a robust evaluation in steps (2) and (3), the split-sample and cross-validation approaches were used. Specifically, 70% of the samples were allocated for training, while the remaining 30% were used for validation. To reduce the impact of random noise, the training process was repeated 30 times, with training and testing datasets randomly selected, and the averaged values of the coefficient of determination (*R*^2^) and the normalized root mean square error (NRMSE) were obtained.

### Principal component regression (PCR) and partial least squares regression (PLSR)

PCR and PLSR methods are common in that they use the concept to reduce data dimensions by extracting components, which are linear combinations of all the independent variables. The most significant difference between PCR and PLSR lies in the principle of component calculation. In PCR, the judgment of components only considers the variance of independent variables (Wold et al., 1987), while in PLSR, the covariance between dependent and independent variables is the criterion to determine components (Wold et al., 2001). The programs of PCR and PLSR were designed based on the “pcr” and “plsr” functions in the “pls” package (Mevik & Wehrens, 2007) and operated in R studio (RStudio Team, 2016), which is an integrated development environment for R project (R Development Core Team, 2008). The “leave-one-out” cross-validation method was performed to select the suitable number of components. The cross-validation result gave a list of root-mean-square errors (*RMSE*) corresponding to different numbers of components. The number of components that give the minimum *RMSE* value of each water quality index was selected to generate the final model.

### Artificial neural network (ANN)

The ANN model consists of input, hidden, and output layers based on a backpropagation framework. The number of neurons was set as the number of wavelengths for the input layer and one neuron in the output layer. Fifteen neurons were used in the hidden layer for nonlinear transformations based on a trial and error method (Zhao et al., 2021, 2022). The “relu” function was selected as the transfer function. The maximum steps for the training of the neural network were fixed at 5000. To avoid overfitting, the early stopping approach was used with a patience of 200 (Paguada et al., 2023). The package “tensorflow” in Python was applied as the core utility for programming (Muñoz-Ordóñez et al., 2018).

### Model evaluation

The following criteria were adopted to evaluate the performance of each model during the prediction using the test datasets acquired by both the desktop and in situ spectrometers. The explanatory power of the models was expressed by the coefficient of determination *R*^2^ calculated using Eq. 1:

1 $$\begin{aligned}&{R}^{2}=1-\frac{SSE}{SST}=1-\frac{\sum {\left({\widehat{y}}_{\mathrm{i}}-{y}_{\mathrm{i}}\right)}^{2}}{\sum {\left({y}_{\mathrm{i}}-\overline{y }\right)}^{2}}\\& (SSE\le SST) { \;\mathrm{or} \;R}^{2}=0 (SSE>SST)\end{aligned}$$

where *SSE* and *SST* represent the sum of squared errors and the total sum of squares, respectively, ${\widehat{y}}_{\mathrm{i}}$ is the calibrated or predicted concentration of a specific water quality index, and *y*_i_ is the corresponding measured concentration; $\overline{y }$ is the mean value of the measured concentrations in the test dataset. *R*^2^ generally distributes between 0 and 1 because *SSE* is usually not higher than *SST*. However, when errors are extremely large, *SSE* can be higher than *SST*. In that case, *R*^2^ is directly assigned 0 to avoid the appearance of a negative value. The accuracy of the models was assessed by the normalized root mean square error calculated from Eq. 2:

2 $$NRMSE=\frac{\sqrt{\sum \frac{{\left({\widehat{y}}_{\mathrm{i}}-{y}_{\mathrm{i}}\right)}^{2}}{n}}}{\overline{y} }$$

where *n* is the number of samples in the corresponding sample set. Higher *R*^2^ and lower *NRMSE* imply higher reliability and accuracy of models.

## Results and discussion

### Determination of the influential wavelengths

**Fig. 4:**
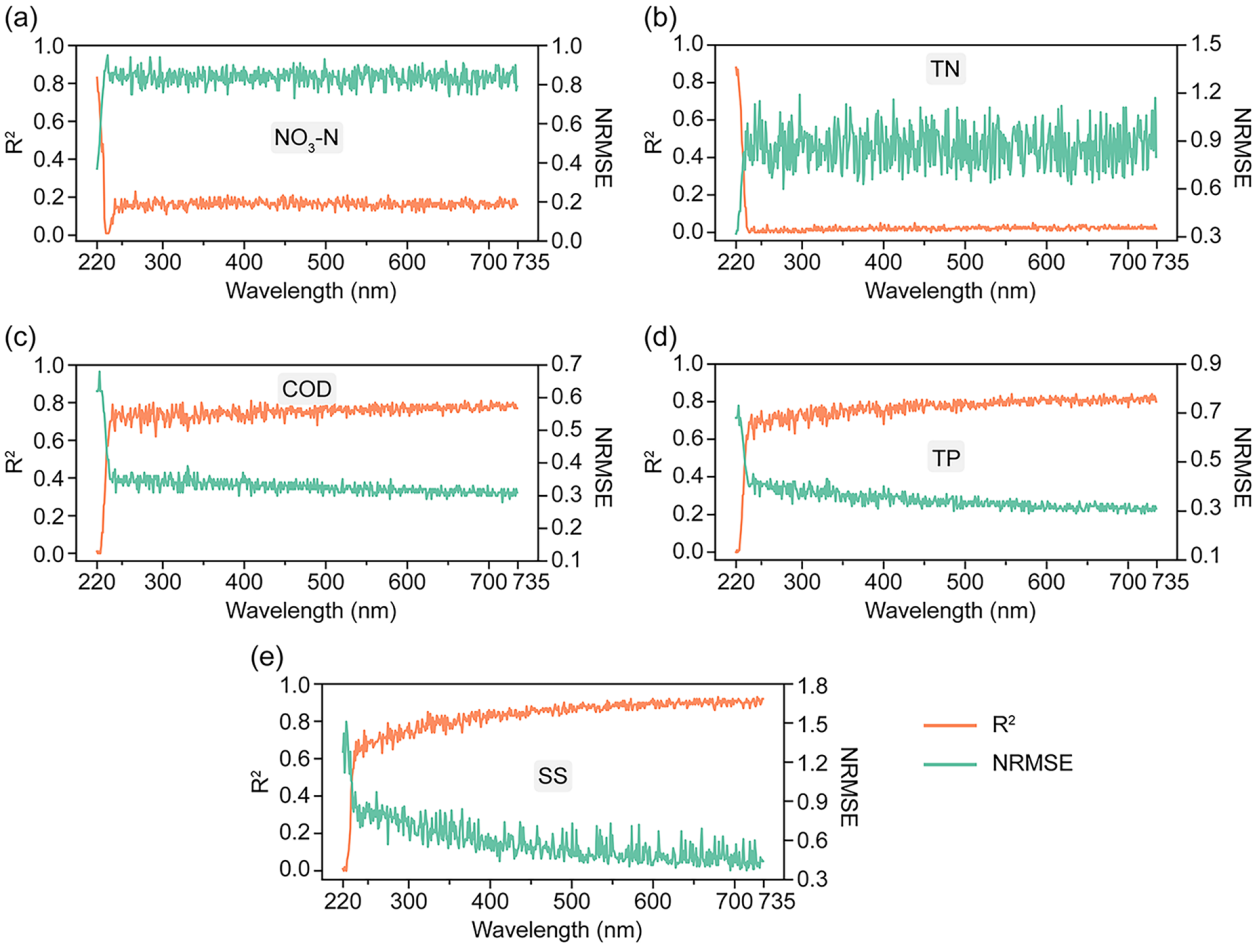
*R*^2^ and NRMSE as functions of wavelength with 1-nm intervals based on simple regression. **a** NO_3_-N, **b** TN, **c** COD, **d** TP, **e** SS

The statistical results of all the water quality parameters are summarized in Table 2. The degree of dispersion of these data can meet the requirements for modeling. The representative UV–Vis spectra recorded by the desktop spectroscopy are presented in Supplementary Fig. 2(a). To determine the wavelengths that have the most impact and select suitable inputs for developing models of each water quality parameter, we analyzed the *R*^2^ and NRMSE values derived from regressions conducted at different wavelengths in the spectra acquired by the desktop spectrometer. Figure 4 shows the *R*^2^ and NRMSE calculated from simple regression results using absorption at each wavelength with 1-nm intervals. For NO_3_-N and TN, the influential wavelengths were confined to the UV range (< 235 nm), with the maximum *R*^2^ and minimum NRMSE observed at 220 nm. This finding is in accordance with the adsorption range of N = O chromophores, which possess an abundance of π electrons. On the contrary, the correlation with absorbance at 220 nm was relatively weak for COD, TP, and SS. As the wavelength increased, the *R*^2^ improved, and the NRMSE decreased. The variations in *R*^2^ and NRMSE for COD and TP tended to be limited over 235 nm, while for SS, the correlation gradually improved as the wavelength increased from 235 nm and the highest *R*^2^ and lowest NRMSE were found near 735 nm, in the red-light range at the end of UV–Vis spectra.

**Table 2:** Statistics on measured water quality data

	NO_3_-N	TN	COD	TP	SS	NH_4_-N	DTP
Min	0.473	1.02	2.30	0.043	2.00	0.003	0.016
Max	9.90	26.4	38.8	1.01	767	16.8	0.19
Mean	2.56	3.84	14.5	0.339	110.7	0.641	0.062
SD	2.26	3.45	8.89	0.225	140.5	2.347	0.035

### Selection of calibration methods

**Fig. 5:**
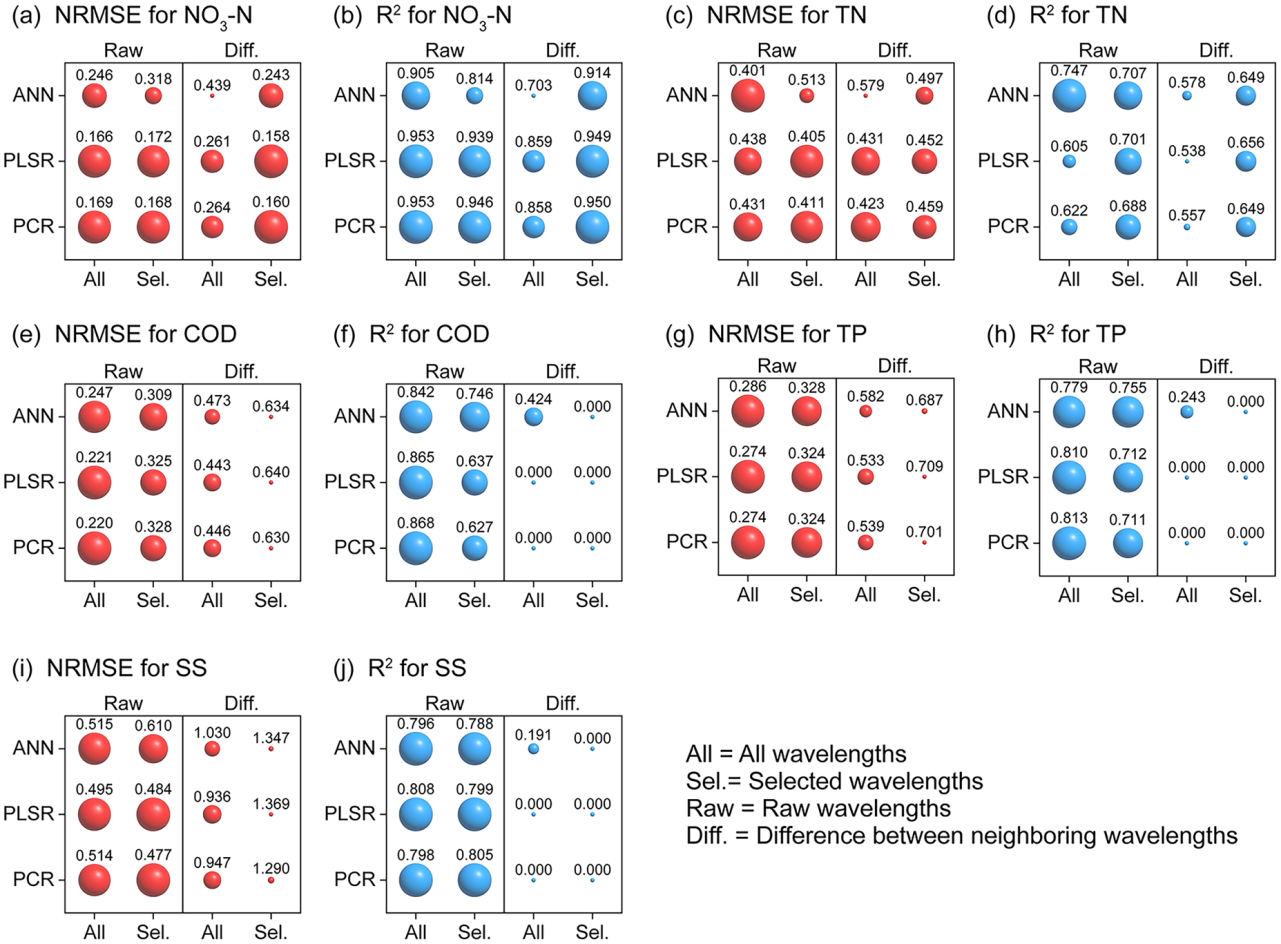
Comparison of different approaches (i.e., different regression models and different wavelength information) to estimate the concentrations of water quality parameters: **a**, **b** NO_3_-N, **c**, **d** TN, **e**, **f** COD, **g**, **h** TP, and **i**, **j** SS. The larger spheres indicate higher *R*^2^ or lower NRMSE values

The *R*^2^ and NRMSE values shown in Fig. 5 were obtained through different calibration methods and wavelength information. This included the use of differential or raw spectra and all or selected wavelengths based on desktop UV–Vis analysis. The results were averaged from 30 randomly repeated training processes for each calibration approach. Supplementary Fig. 3 demonstrates that the standard deviations of *R*^2^ and NRMSE obtained during the repeated training processes were limited, ensuring the reliability and repeatability of the preliminary model evaluation results.

The differential spectra were calculated by subtracting the absorbance values between neighboring wavelengths (Supplementary Fig. 2(b)). The selected wavelengths comprised the 3–5 most influential wavelengths (with 5-nm interval) identified in Fig. 4. The calibration approach that yielded the lowest NRMSE and a high *R*^2^ exceeding 0.65 for each water quality parameter was deemed the most effective and summarized in Table 3. In Fig. 5(a)–(d), both PCR and PLSR exhibited comparable goodness, with *R*^2^ reaching approximately 0.85 − 0.95 for NO_3_-N and 0.5 − 0.7 for TN, and NRMSE ranging from ~ 0.2 for NO_3_-N to a slightly higher value of ~ 0.4‒0.5 for TN. The differences in *R*^2^ and NRMSE were insignificant for both NO_3_-N and TN between PLSR and PCR for each type of wavelength subset. The nonlinear ANN method slightly underperformed compared to PCR and PLSR for NO_3_-N but showed better applicability for TN when utilizing raw absorption spectra across the entire wavelength range, with *R*^2^ reaching ~ 0.7 − 0.75. In general, PCR and PLSR achieved low NRMSE values averaging ~ 0.16–0.26, indicating promising performance for laboratory-calibrated models predicting NO_3_-N for in situ applications. However, both methods yielded higher NRMSEs of ~ 0.4 for TN estimation. PLSR exhibited relatively better performance for NO_3_-N estimation when utilizing the difference in selected wavelengths, while the ANN method with raw data inputs proved more suitable for TN (Table 3). It is evident that the use of the difference data eliminated the elevation of the spectrum baseline primarily caused by the suspended sediments, thereby improving the accuracy of NO_3_^−^ estimation compared with results obtained using raw spectra. However, TN comprises not only soluble nitrate but also a small amount of insoluble matter adsorbed by suspended particles; thus, the raw wavelengths without baseline correction showed slightly improved performance. This phenomenon was also pronounced in the COD, TP, and SS estimations when comparing results derived from raw and differential spectra (Fig. 5(e)–(j)). For COD, TP, and SS, the average *R*^2^ values obtained using raw wavelengths reached ~ 0.8, significantly higher than the values of approximately 0.2–0.3 derived from differential wavelengths. Similarly, low NRMSE values of approximately 0.2–0.3 for COD and TP and approximately 0.4–0.6 for SS were achieved using raw spectra, which outperformed the results obtained using differential spectra (approximately 0.45–0.7 for COD and TP, and > 1 for SS). Furthermore, the pronounced correlation between SS and COD and SS and TP and the weak correlation between SS and dissolved total phosphorus (DTP) (Table 4) indicate that suspended particles serve as carriers of organic compounds and phosphorous. These findings explain why COD, TP, and SS can only be well estimated using raw wavelengths without baseline correction. Additionally, using all wavelengths for calibration model creation proved preferable for COD and TP, resulting in lower NRMSE values and higher *R*^2^. When using all wavelengths and raw spectra, the PCR model exhibited a slightly lower NRMSE and a slightly higher *R*^2^ than the PLSR model for COD calibration (Fig. 5(e) and (f)), suggesting that the use of the PCR model is preferable. For TP, both PLSR and PCR models yielded the same NRMSE value, and their *R*^2^ values were similar (Fig. 5(g) and (h)). Therefore, it is suggested to consider both the PLSR and PCR models as candidates for further validation. In the case of SS, we also recommend two models for further validation after comparing the calculated results of NRMSE and *R*^2^. As shown in Fig. 5(i) and (j), the PCR model based on the selected wavelengths of raw spectra exhibits the lowest NRMSE of 0.477, while the PLSR model utilizing all wavelengths of raw spectra shows the highest *R*^2^.

**Table 3:** Suggested approaches for estimating the concentration of water quality parameters

**Parameter**	**Regression model**	**Raw or difference**	**Wavelength**
**NO**_**3**_**-N**	PCR PLSR	Difference	Selected wavelengths (220, 225, 230, 235 nm)
**TN**	ANN	Raw	All wavelengths
**COD**	PCR	Raw	All wavelengths
**TP**	PCR	Raw	All wavelengths
	PLSR		
**SS**	PCR	Raw	Selected Wavelengths (725, 730, 735 nm)
	PLSR		All wavelengths

**Table 4:** Pearson’s correlation coefficients among TP, PP, DTP, COD, and SS

TP	1.000				
PP	0.990	1.000			
DTP	0.340	0.204	1.000		
COD	0.959	0.964	0.228	1.000	
SS	0.875	0.906	0.033	0.873	1.000
	TP	PP	DTP	COD	SS

*TP* = total phosphorus

*DTP* = dissolved total phosphorus

*PP* = particle phosphorus

### Water quality estimation results

**Fig. 6:**
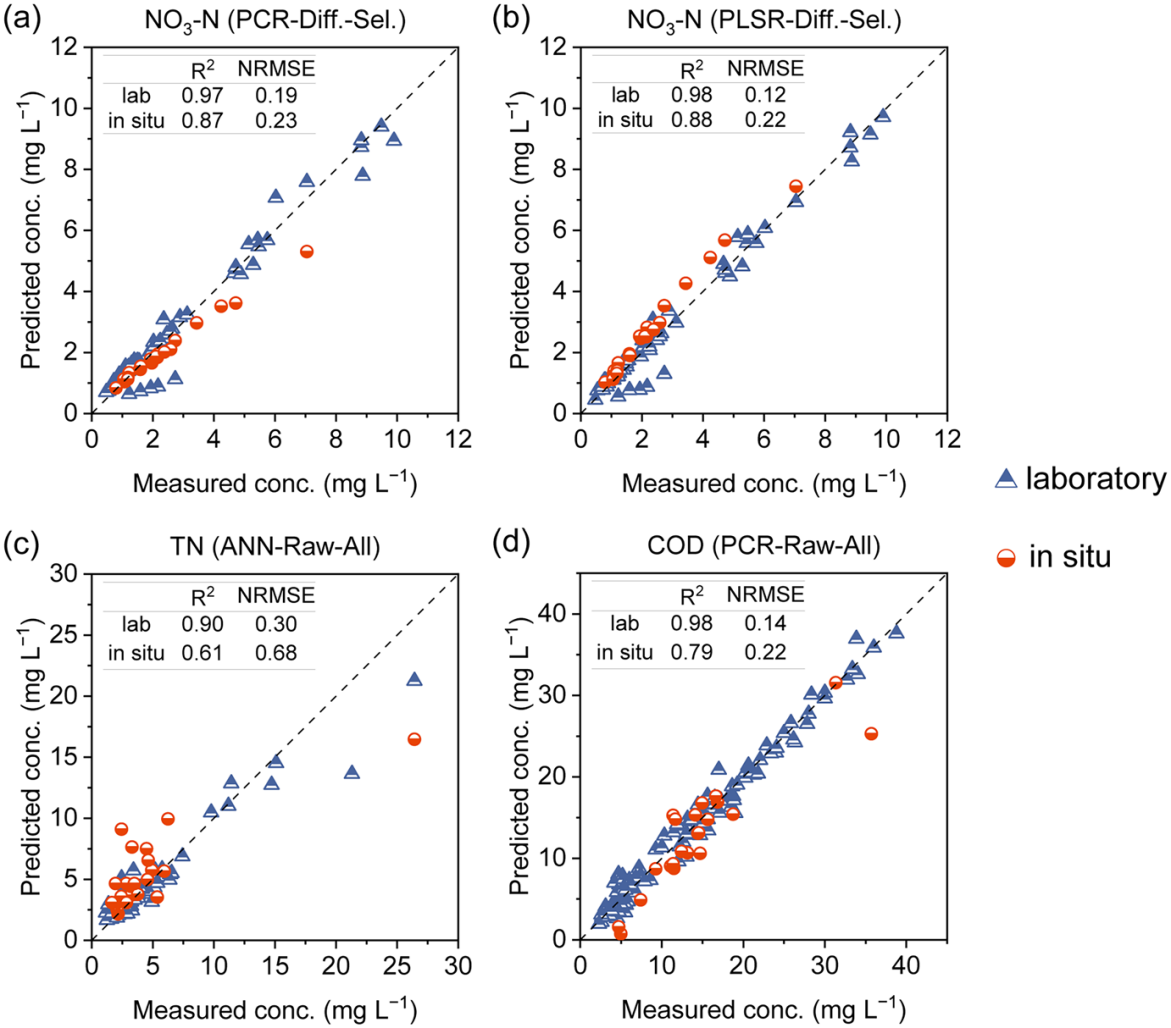
Validation results of the proposed approaches (listed in Table 3) for the estimation of **a, b** NO_3_-N, **c** TN, and **d** COD

**Fig. 7:**
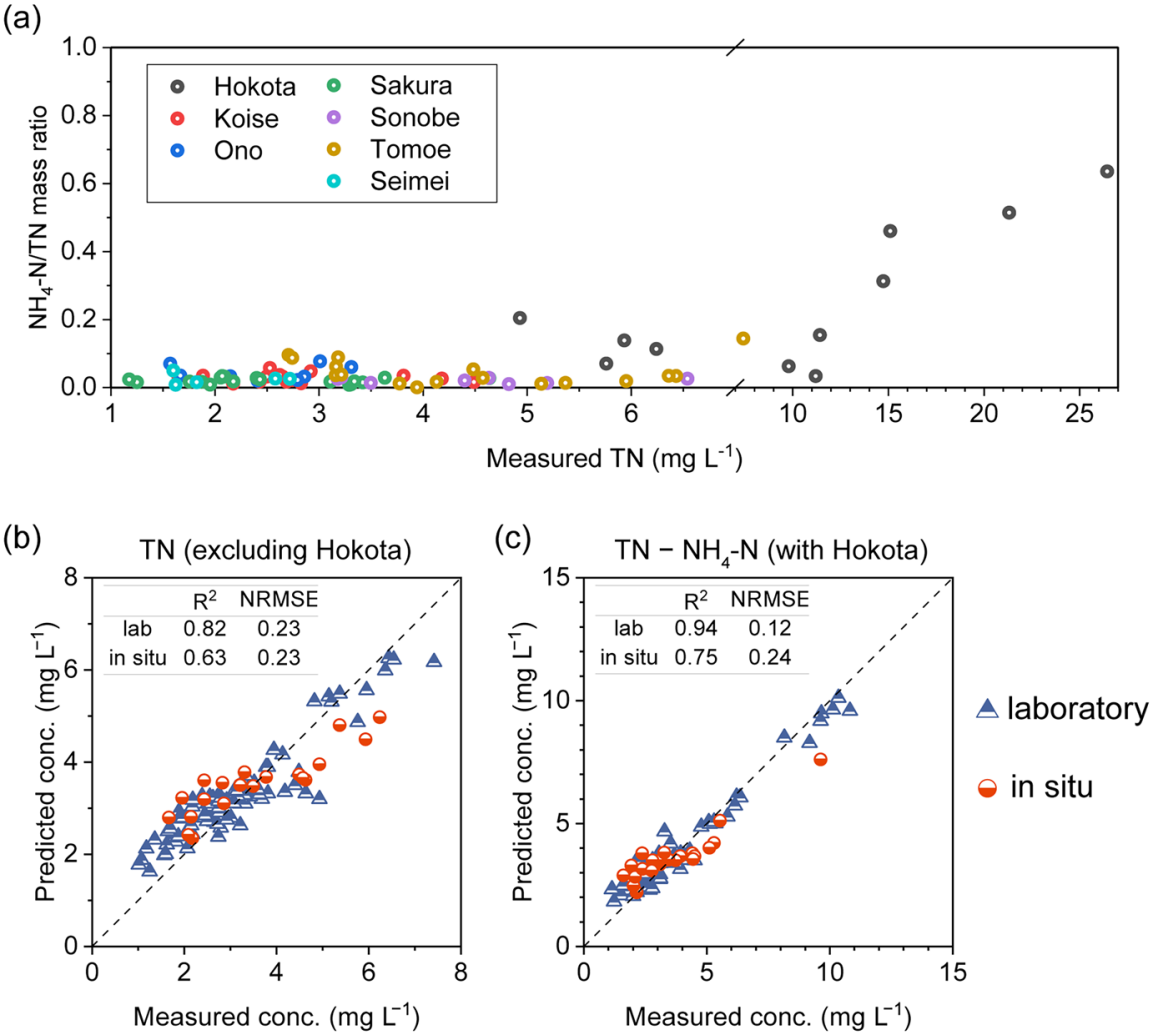
**a** Diagram of NH_4_-N/TN mass ratio versus TN concentration. Validation results for estimating **b** TN with the data points from the Hokota River excluded and **c** TN − NH_4_-N using all the data points

Figure 6 shows the validation results of the proposed approaches for estimating NO_3_-N, TN, and COD. The laboratory-calibrated models for NO_3_-N and COD exhibited excellent validation results. In the case of NO_3_-N, the PLSR model, using differential spectra and selected wavelengths, demonstrated slightly improved performance compared to the PCR model. The *R*^2^ value for NO_3_-N estimation using the desktop UV–Vis data reached an impressive 0.98, with a relatively low NRMSE of 0.12 (Fig. 6(a) and (b)). Furthermore, the PLSR model showed reliable performance in estimating NO_3_-N based on in situ spectrum data, with *R*^2^ and NRMSE values of 0.88 and 0.22, respectively. Similar performance was observed for COD estimation using the PCR model, utilizing raw spectra and all wavelengths (Fig. 6(d)). The *R*^2^ and NRMSE values obtained using the desktop UV–Vis data were 0.98 and 0.14, respectively. In comparison, the in situ data yielded *R*^2^ and NRMSE values of 0.79 and 0.22, respectively. These results strongly support the practical application of the prediction models calibrated using desktop UV–Vis spectra, with high accuracy ensured. The TN calibration and prediction were accurate for concentrations below 15 mg L^−1^, but displayed decreased accuracy at higher concentration levels (Fig. 6(c)). Consequently, the NRMSEs exceeded 0.3 for calibration and reached 0.68 for prediction using in situ data. It was observed from the experimental data that several water samples, particularly from the Hokota River, contained a significant proportion of NH_4_^+^. The N–H σ-bond in NH_4_^+^ is minimally responsive to UV–Vis light excitation (Fig. 7(a)). Therefore, the more significant errors observed in predicting high concentrations of TN compared to NO_3_-N concentrations can be attributed to the interference of optically inert NH_4_^+^. By excluding the data points from the Hokota River, the overall errors were notably reduced, resulting in NRMSEs of 0.23 for both the laboratory and in situ data (Fig. 7(b)). This explanation was further corroborated by constructing another calibration model to estimate organic and inorganic nitrogen concentrations, excluding NH_4_-N, obtained by subtracting NH_4_-N concentration from TN concentration (TN–NH_4_-N), using the same modeling approach. As shown in Fig. 7(c), a significantly improved agreement was observed compared to the validated TN results (Fig. 6(c)), with *R*^2^ values reaching up to 0.94 and 0.75 for laboratory data and in situ data, respectively. The NRMSEs also resulted in low values (0.12 and 0.24 for laboratory and in situ data, respectively), approaching the high accuracy achieved in the NO_3_-N validation.

**Fig. 8:**
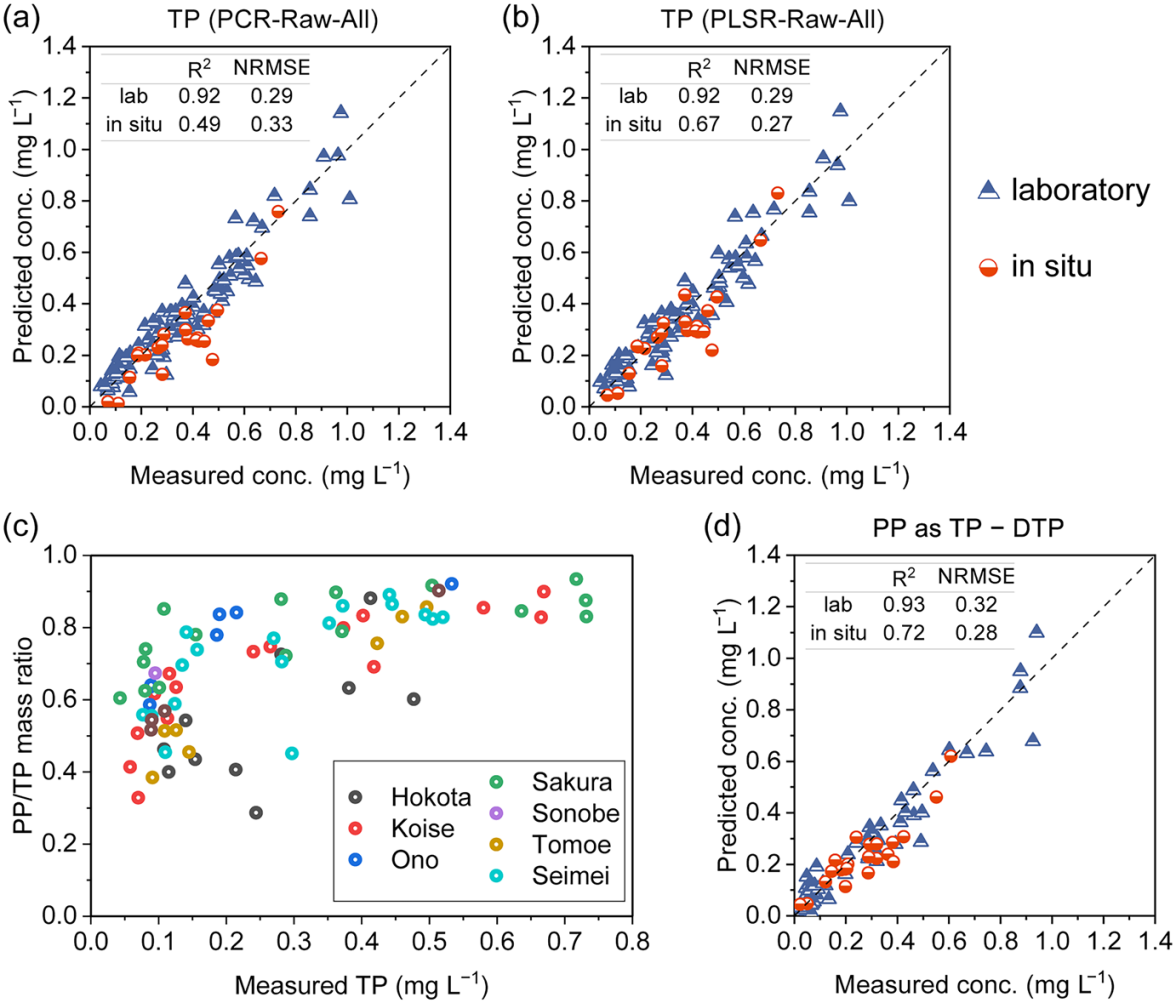
Validation results of the proposed approaches for the estimation of TP: **a** PCR model, raw spectra, and all wavelengths; **b** PLSR model, raw spectra, and all wavelengths. **c** Diagram of PP/TP mass ratio versus TP concentration. **d** Validation result for the estimation of PP (as TP − DTP)

We compared two candidate approaches for TP validation (Fig. 8(a) and (b)). Although PCR and PLSR yielded the same *R*^2^ and NRMSE values for the laboratory data, PLSR is deemed preferable due to its satisfactory agreement between measured and predicted concentrations, with a higher *R*^2^ (0.67) and lower NRMSE (0.27) compared to the PCR model for in situ data. Previous research involving in situ UV–Vis spectroscopy indicated that TP prediction is less accurate than other water quality parameters, such as NO_3_–N (Etheridge et al., 2014). However, our results suggest that TP can be well calibrated and predicted using all wavelengths as inputs for the PLSR method. The improved outcomes in our study can be attributed to the dominant presence of particulate phosphorous (PP) in stream water (Fig. 8(c)). The observed optical response of phosphorous likely arises from electron transitions between bonding and antibonding orbitals in adsorbed organic phosphorus on suspended particles or intrinsic excitations in phosphorous-containing inorganics such as biogenic apatite, which are commonly found in soils (Kizewski et al., 2011; Rulis et al., 2004). The noticeable correlation between SS and TP (Table 4) further suggests that suspended particles serve as carriers of phosphorous. To investigate the relationship between phosphorous and suspended particles in more detail, we calibrated a model to estimate PP using the same PLSR approach employed for TP. As a result, similar performance was confirmed for the validation of both the laboratory and in situ data (Fig. 8(d)). These findings explain why TP can be well predicted based on UV–Vis absorbance spectra.

**Fig. 9:**
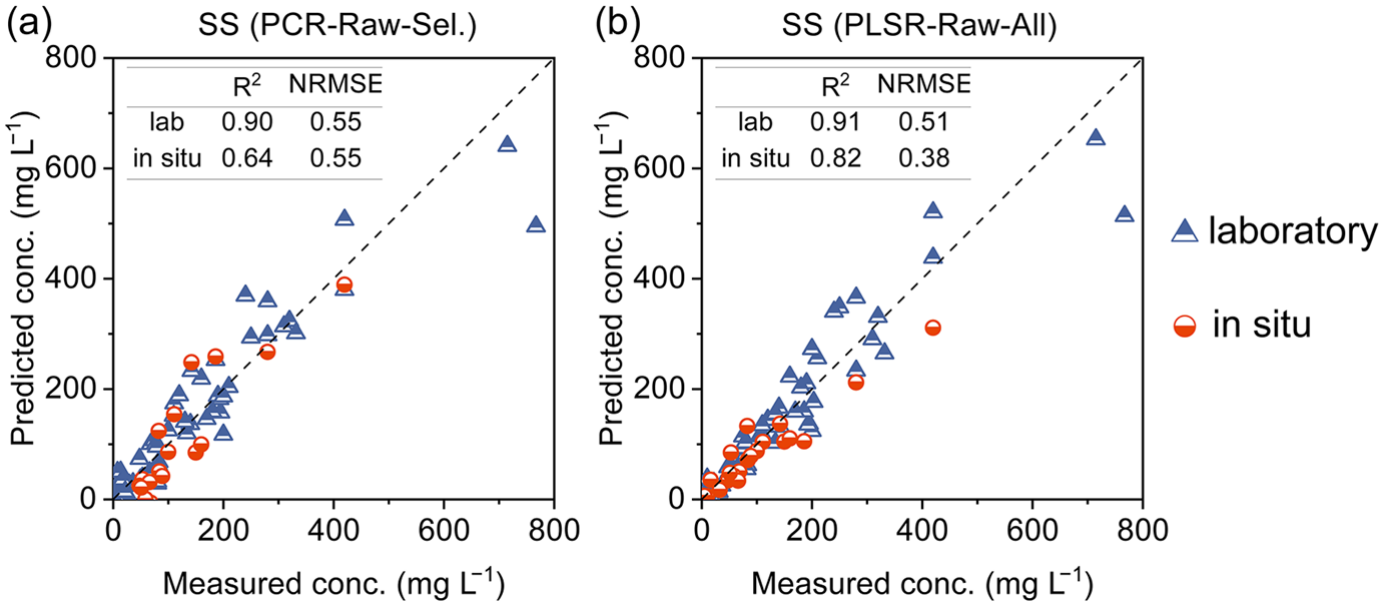
Validation results of the proposed approaches for the estimation of SS. **a** PCR model, raw spectra, and selected wavelengths; **b** PLSR model, raw spectra, and all wavelengths

We also compared two candidate approaches for estimating SS and confirmed that the PLSR model, using raw spectra and all wavelengths, outperformed the PCR model, which employed raw spectra and selected wavelengths, in predicting SS based on in situ data, although they exhibited similar validation results for the laboratory data (Fig. 9). We achieved high *R*^2^ values of 0.91 and 0.82 for the laboratory and in situ data, respectively, while the NRMSE values were 0.51 and 0.38, relatively inferior to other water quality parameters. It was observed that the absolute validation errors of SS increased with concentration. The optical response of suspended solids is influenced by various factors, such as light absorption caused by organic compounds and metal ions, particle size-dependent Mie scattering, and diffraction (see Supplementary Fig. 4 for particle size analysis) (Berho et al., 2004). Due to the complexity involved, the multiple linear relationships between SS concentrations and absorbances at different wavelengths may be partially distorted, thereby reducing accuracy during calibration and validation processes to some extent. Nevertheless, the developed regression and neural network models show promise for monitoring selected river water quality parameters over a wide range of concentrations under both low-flow and flooding conditions. Further studies should focus on enhancing anti-interference properties under specific conditions, such as runoff events and accidental water pollution.

Finally, to enable a more comprehensive comparison, we assessed the performance of all selected best models based on the rating criteria proposed in a previous study (Moriasi et al., 2007). As shown in Supplementary Table 1, for both water quality parameters predicted using the desktop and in situ spectral data, the majority of obtained *R*^2^ and NRMSE values fall within the categories of “very good” and “good.” Although the prediction performance of in situ spectral data for TN was slightly lower, it still reached the “very good” level after eliminating the influence of NH_4_-N. These results demonstrate that our proposed models should meet the basic requirements for practical applications.

## Conclusions

We investigated a framework integrating laboratory-based (desktop) and in situ UV–Vis spectroscopy, employing statistical regression and machine learning technologies. We developed prediction models for water quality parameters such as NO_3_-N, TN, COD, TP, and SS in eutrophic rivers draining from watersheds with varying land use characteristics. Our results revealed that NO_3_-N and COD were accurately estimated using PLSR and PCR models, respectively, with selected wavelengths from the UV–Vis spectra as model inputs. For the calibration and prediction of TN, the ANN model was found to be more effective when the NH_4_-N concentration was relatively low. NO_3_-N was the only parameter that required the difference of spectra as model inputs. TP and SS were predicted effectively by PLSR and PCR models, respectively, by employing all wavelengths. The successful prediction of TP was primarily attributed to the presence of photo-responsive phosphorus species adsorbed onto suspended solids. The proposed framework offers an alternative approach for the efficient and less laborious development and application of prediction models for river water quality monitoring, utilizing in situ UV–Vis spectroscopy. Furthermore, this approach can potentially facilitate the adoption of advanced online technology for water quality monitoring in the realm of water pollution control.

### Supplementary Information

Below is the link to the electronic supplementary material.

Supplementary file1 (DOCX 2.84 MB)

## Data Availability

The datasets generated in this study are available from the corresponding author upon reasonable request.
