# Supplementary material for: Development of statistical regression and artificial neural network models for estimating nitrogen, phosphorus, COD, and suspended solid concentrations in eutrophic rivers using UV–Vis spectroscopy
Source: Environ Monit Assess. 2023 Aug 31;195(9):1114. doi: 10.1007/s10661-023-11738-0 (PMC10468949; doi:10.1007/s10661-023-11738-0)
Supplement: Supplementary file 1 — Supplementary file1 (DOCX 2.84 MB) [file 10661_2023_11738_MOESM1_ESM.docx]

***Supplementary Information***

**Development of statistical regression and artificial neural network models for estimating nitrogen, phosphorus, COD, and suspended solid concentrations in eutrophic rivers using UV-Vis spectroscopy**

Yanping Lyu ^1^, Wenpeng Zhao ^1,2*^, Tsuyoshi Kinouchi ^1*^, Tadahiro Nagano ^3^, Shigeo Tanaka ^3^

^1^ Department of Transdisciplinary Science and Engineering, Tokyo Institute of Technology, 4259 Nagatsuta-cho, Midori-ku, Yokohama, Kanagawa 226-8503, Japan.

^2^ College of Hydraulic Science and Engineering, Yangzhou University, Yangzhou 225009, China.

^3^ Civil Engineering and Eco-Technology Consultants Co., Ltd, 2-23-2 Higashi-Ikebukuro, Toshima-ku, Tokyo 170-0013, Japan.

* Correspondence: Tsuyoshi Kinouchi, kinouchi.t.ab@m.titech.ac.jp

Wenpeng Zhao, wppzhao@gmail.com


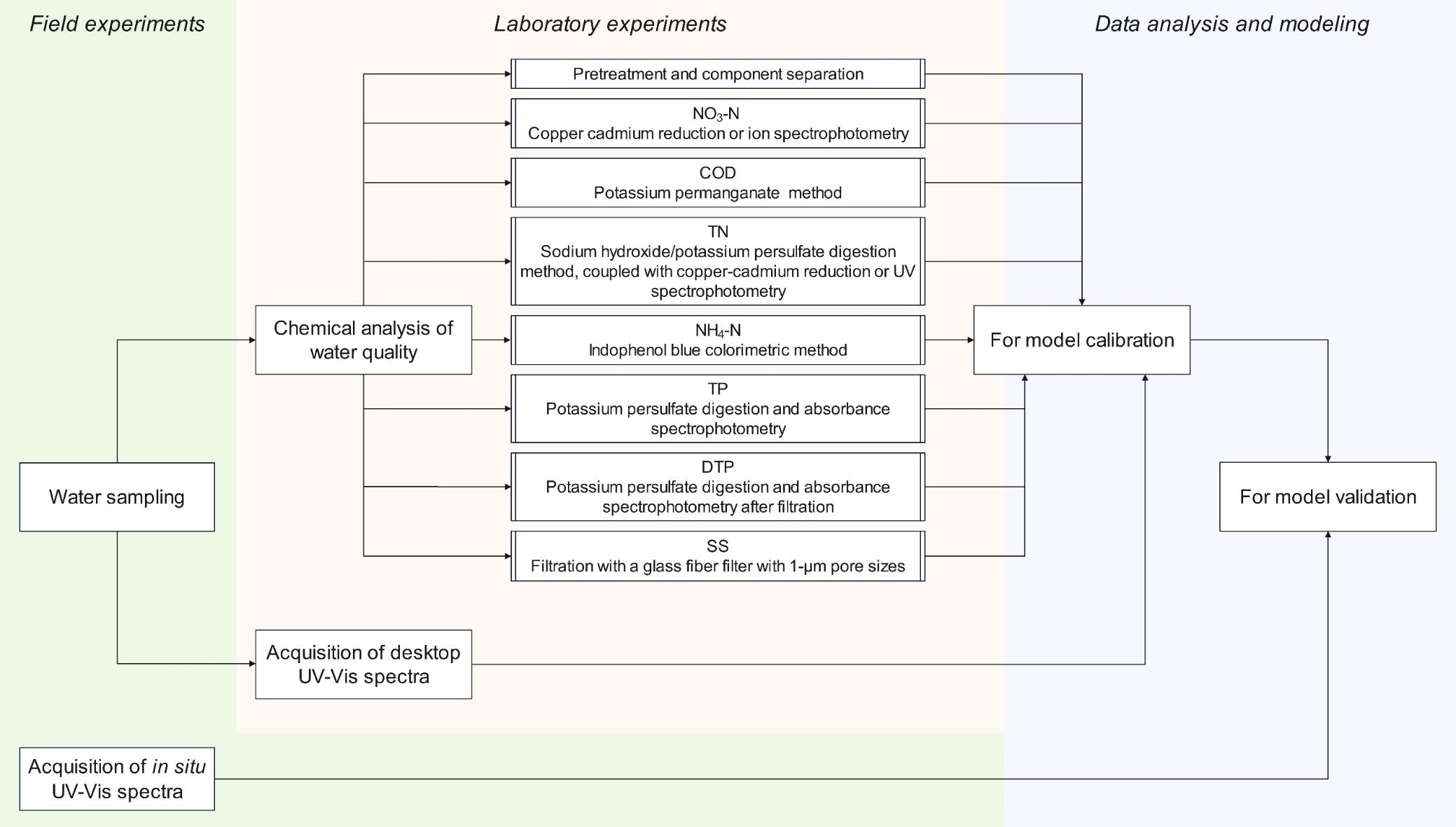


**Supplementary Fig. 1** Flowchart of the experiments for the acquisition of the water quality data and UV-Vis spectra


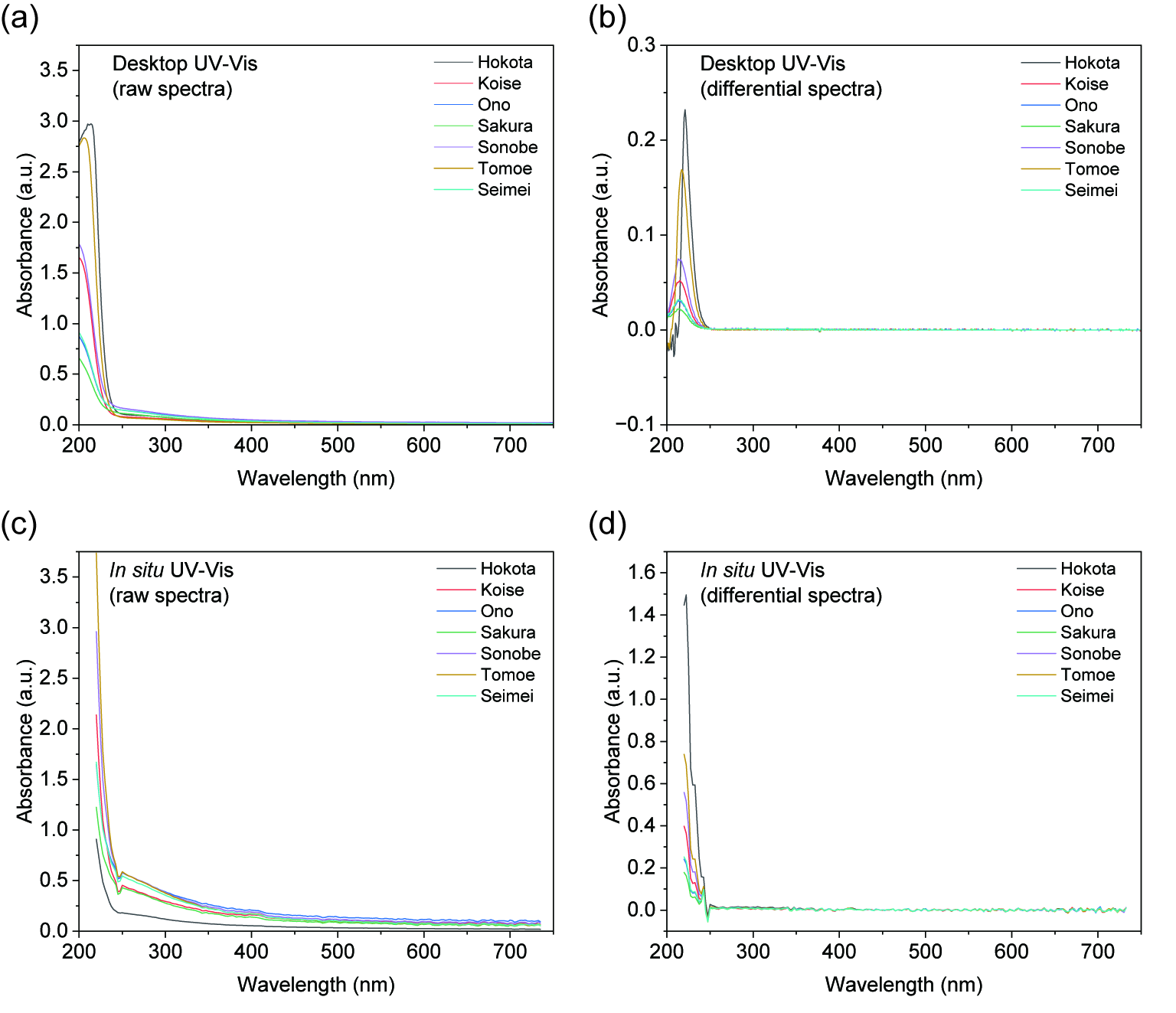


**Supplementary Fig. 2** Representative UV-Vis spectra recorded using a desktop spectroscopy (a, b) and a in situ spectroscopy (c, d). (a) and (c) are the raw spectra. (b) and (d) are the differential spectra.


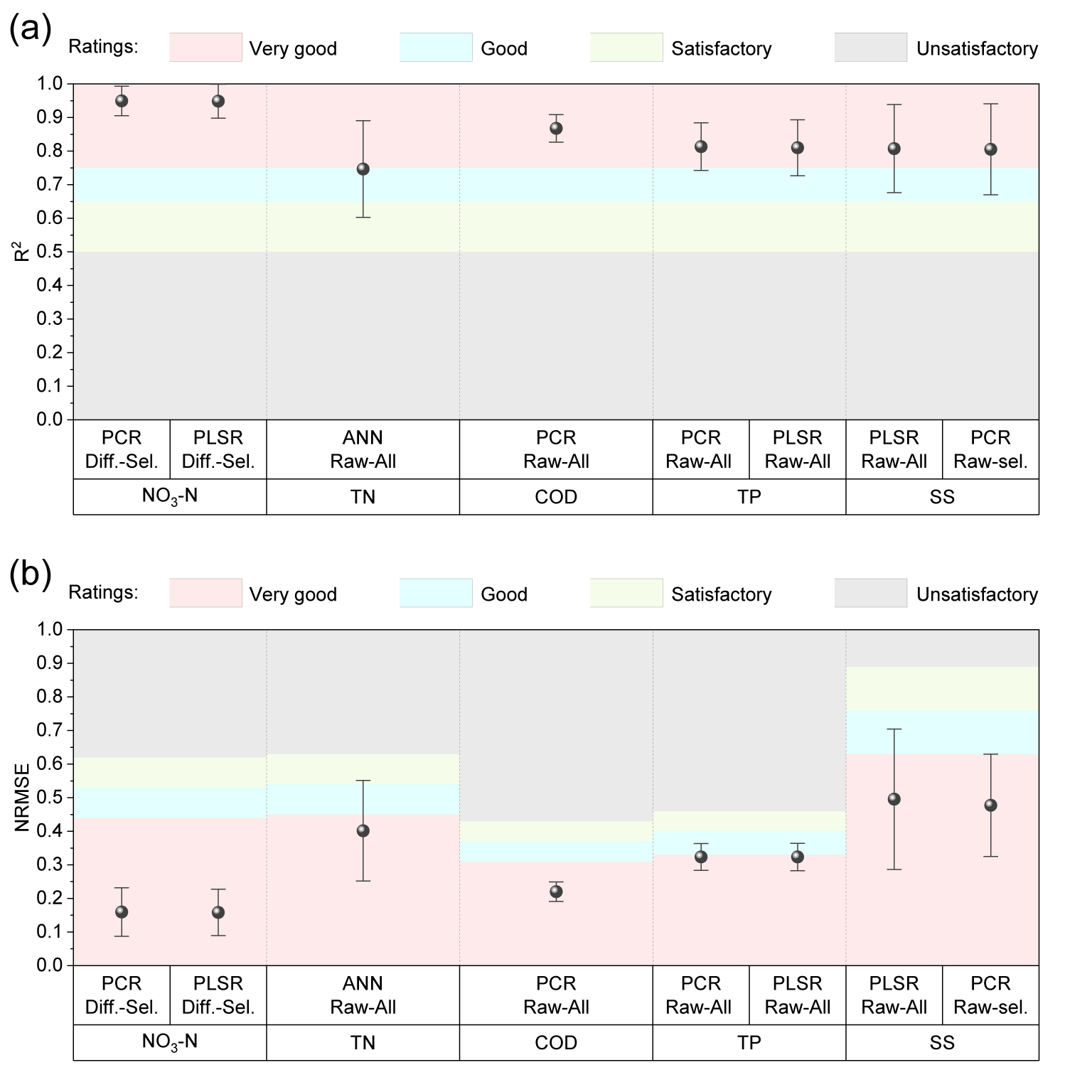


**Supplementary Fig. 3** The reproducibility of model calibrations. The error bars represent the standard deviations of (a) R^2^ and (b) NRMSE across 30 randomly repeated training processes during the model calibrations based on the desktop UV-Vis spectra. All the performance rating guidelines for R^2^ and NRMSE are originally proposed by Moriasi et al. (2007). The variations of R^2^ and NRMSE values were within the ‘satisfactory’ range across 30 randomly repeated training processes.


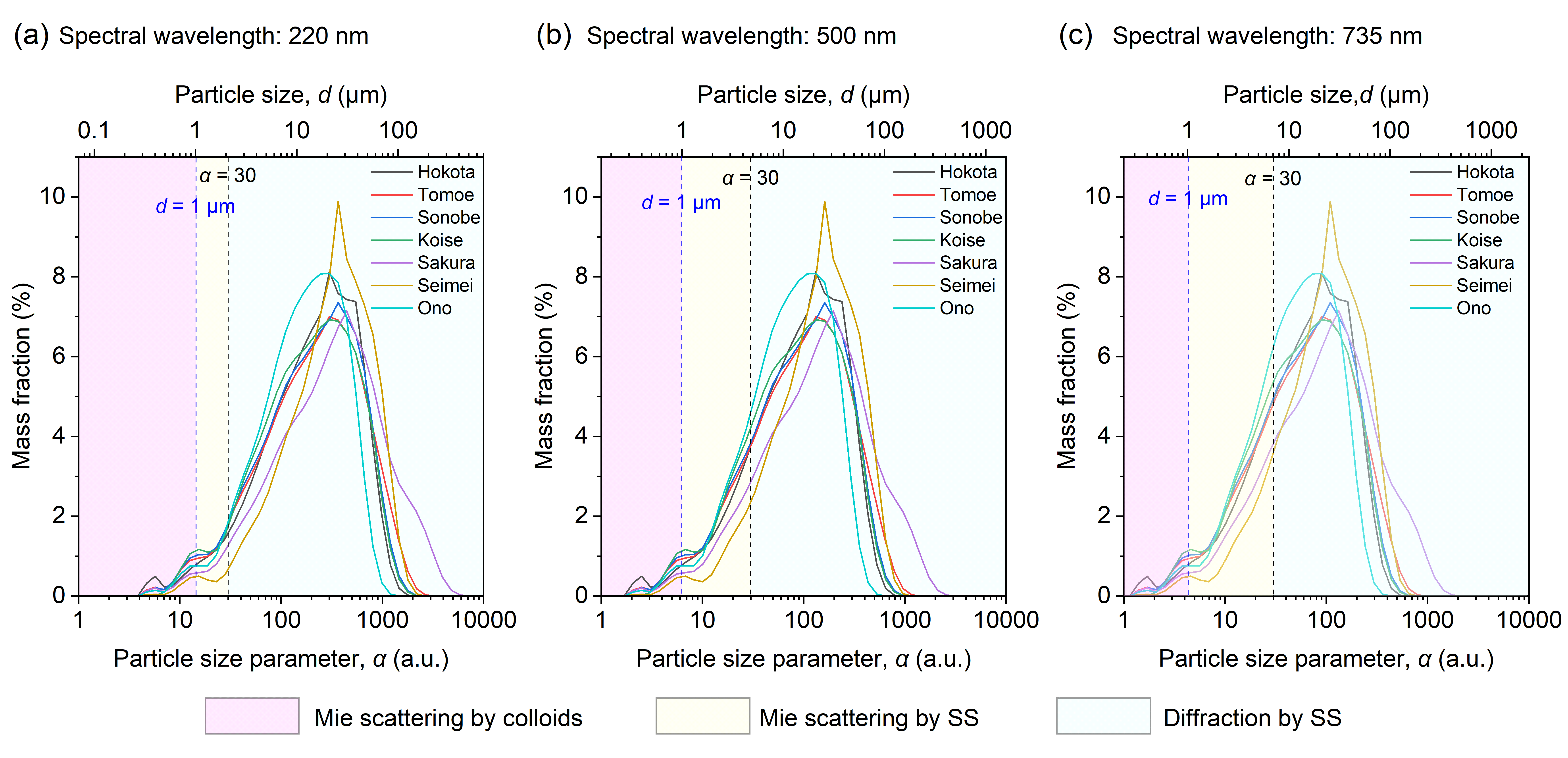


**Supplementary Fig. 4** Average distribution of particle size of the suspended solids and colloids in different rivers and parameter α calculated at spectral wavelengths of (a) 220 nm, (b) 500 nm, and (c) 735 nm in the rivers in Kasumigaura Lake watershed. The particle sizes were measured using a Shimazu SALD-3000J analyzer. The interactions between a light beam and particles primarily depend on the ratio between the particle size (diameter *d*) to the wavelength (*λ*) of the light beam. Previous studies (Azema et al. 2002; Berho et al. 2004; Kleitz and Boulaud 1995) have commonly used a size parameter *α* is generally used to distinguish between the domains of scattering and diffraction, which can be calculated using the following equation:

$$\alpha=\frac{\pi d}{\lambda}$$

For *α* < 0.3 (indicating a particle size much smaller than the wavelength), the optical model of Rayleigh diffraction can be utilized. In this case, the scattered light is equally distributed in the forward and backward directions. For *α* > 30 (indicating a particle size larger than the wavelength), diffraction laws can be applied, and the diffused light is predominantly concentrated in front of the particles. For 0.3 < *α* < 30, (indicating a particle size close to the wavelength), the Lorenz-Mie theory (Mie scattering) can be employed. In the present study, the incident light from in situ UV-Vis spectrometer covers a wide range of wavelengths, from 220 to 735 nm. Therefore, we selected 220, 500, and 735 nm as examples to calculate the distributions of the size parameter α under different wavelength conditions. As depicted in this figure, all water samples exhibited three domains for particle distribution: Mie scattering by colloids (*d* < 1 μm, *α* < 30), Mie scattering by SS (*d* > 1 μm, *α* < 30), and diffraction by SS (*d* > 1 μm, *α* > 30). For SS, diffraction was dominant at all wavelengths, although the proportion of Mie scattering slightly increased with longer wavelengths.

**Supplementary Table 1.** Evaluation results of the final models based on the suggested model evaluation guidelines.

| Parameter | | NO_3_-N | TN | COD | TP | SS |
| --- | --- | --- | --- | --- | --- | --- |
| Model | | PLSR-Diff.-Sel. | ANN-Raw-All | PCR-Raw-All | PLSR-Raw-All | PLSR-Raw-All |
| R^2^ | Lab | 0.98 (VG) | 0.90 (VG) | 0.98 (VG) | 0.92 (VG) | 0.91 (VG) |
|  |  |  | 0.82 (VG) * |  | 0.93 (VG) ** |  |
|  | In situ | 0.88 (VG) | 0.61 (S) | 0.79 (VG) | 0.67 (G) | 0.82 (VG) |
|  |  |  | 0.63 (S) * |  | 0.72 (G) ** |  |
|  | Ratings | *** VG: (0.75, 1.00]; G: (0.65, 0.75]; S: (0.50, 0.65]; U: [0, 0.50] | | | | |
| NRMSE | Lab | 0.12 (VG) | 0.30 (VG) | 0.10 (VG) | 0.29 (VG) | 0.51 (VG) |
|  |  |  | 0.23 (VG) * |  | 0.27 (VG) ** |  |
|  | In situ | 0.22 (VG) | 0.68 (U) | 0.22 (VG) | 0.33 (VG) | 0.38 (VG) |
|  |  |  | 0.23 (VG) * |  | 0.28 (VG) ** |  |
|  | Ratings | VG: [0.00, 0.44]  G: (0.44, 0.53]  S: (0.53, 0.62]  U: (0.62, +∞) | VG: [0.00, 0.45]  G: (0.45, 0.54]  S: (0.54, 0.63]  U: (0.63, +∞) | VG: [0.00, 0.31]  G: (0.31, 0.37]  S: (0.37, 0.43]  U: (0.43, +∞) | VG: [0.00, 0.33]  G: (0.33, 0.40]  S: (0.40, 0.46]  U: (0.46, +∞) | VG: [0.00, 0.63]  G: (0.63, 0.76]  S: (0.76, 0.89]  U: (0.89, +∞) |

* Without the samples collected from the Hokota River

** PP as TP−DTP

*** VG, G, S, and U represent very good, good, satisfactory, and unsatisfactory, respectively. All the rating guidelines are originally proposed by Moriasi et al. (2007).

**References**

Azema, N., Pouet, M.-F., Berho, C., & Thomas, O. (2002). Wastewater suspended solids study by optical methods. *Colloids and Surfaces A: Physicochemical and Engineering Aspects*, *204*(1), 131–140. https://doi.org/10.1016/S0927-7757(02)00006-7

Berho, C., Pouet, M.-F., Bayle, S., Azema, N., & Thomas, O. (2004). Study of UV–vis responses of mineral suspensions in water. *Colloids and Surfaces A: Physicochemical and Engineering Aspects*, *248*(1), 9–16. https://doi.org/10.1016/j.colsurfa.2004.08.046

Moriasi, D. N., Arnold, J. G., Van Liew, M. W., Bingner, R. L., Harmel, R. D., & Veith, T. L. (2007). Model Evaluation Guidelines for Systematic Quantification of Accuracy in Watershed Simulations. *Transactions of the ASABE*, 50, 885–900. https://doi.org/10.13031/2013.23153

Kizewski, F., Liu, Y.-T., Morris, A., & Hesterberg, D. (2011). Spectroscopic Approaches for Phosphorus Speciation in Soils and Other Environmental Systems. *Journal of Environmental Quality*, *40*(3), 751–766. https://doi.org/10.2134/jeq2010.0169

Kleitz, A., & Boulaud, D. (1995). Granulométrie des particules en mouvement et des aérosols.

*Mesures physiques*. https://doi.org/10.51257/a-v1-r2360
